# Copper content of staple seeds and grains grown in Kanam local government area, Nigeria

**DOI:** 10.1186/2193-1801-2-373

**Published:** 2013-08-06

**Authors:** Kiri Hashimu Jaryum, Zebulon Sunday Chibundo Okoye, Barbara Stoecker

**Affiliations:** Department of Nutritional Sciences, College of Human Science, Oklahoma State University, Stillwater, OK 74078 USA; Department of Biochemistry, Faculty of Medical Sciences, University of Jos, PMB, 2084 Jos, Nigeria

**Keywords:** Grains, Copper, Kanam, Functions, Contents

## Abstract

Copper is a trace mineral that plays an important role in human metabolism, largely because it allows many critical enzymes to function properly. Little is known of copper content of Nigerian foods. In this paper, copper contents of several typical Nigerian foods were determined. The samples were collected from Kanam of Plateau State in central region of Nigeria. The samples were wet-ashed according to the protocol of Hill et al. (Anal Chem 55:2340–2342, 1986).Concentrations of copper in the samples was determined using inductively coupled-mass spectrophotometry. There is, for each crop, a wide variation in copper content. The highest copper content was found in cowpea, Vigna unguiculata (16.95 μg/g of dry weight). Lowest copper content was found in white maize and in yellow maize (Zea mays), with values 1.23 μg/g and 1.38 μg/g of dry weights, respectively. Other foods, such as white sorghum, red sorghum (Sorghum bicolor), millet (Pennisetum glaucum), and groundnuts (Arachis hypogea) had copper contents varying from 2.22 to 11.81 μg/g. These values are well below the supplemental values of 50 mg/day that could interfere with zinc absorption. Thus, among the staple foodstuffs of the areas sampled, cowpea appears to be the richest source of dietary copper followed by groundnut while the two maize varieties are the poorest.

## Introduction

Copper is an essential trace mineral for both physical and mental health. Although Hippocrates is said to have prescribed copper compounds to treat diseases as early as 400 B.C. (Turnlund 2006), the essentiality of copper for man was not recognized until 1928 when Hart et al. (1928) showed copper to be essential for erythropoiesis in rats fed a milk-based diet. Current research suggests that most of the copper in living organisms plays the role of cofactor for specific enzymes and electron transport proteins involved in energy or antioxidant metabolism (Linder & Hazegh-Azam 1996). These functions of copper are possible because of the ability of copper to easily accept and donate electrons forming cuprous (Cu^+^) and cupric (Cu^2+^) ions. Cytochrome *c* oxidase is one of the copper-dependent enzymes. It plays a critical role in the cellular energy production by catalyzing the reduction of molecular oxygen (O_2_) to water (H_2_O). This reaction generates an electrical gradient that is used by the mitochondria to create the energy-storing molecule, ATP (Uauy et al. 1998). Another copper-containing enzyme of vital function is copper-zinc superoxide dismutase, Cu/Zn SOD. This enzyme functions as an antioxidant by catalyzing the conversion of superoxide radicals [free radicals or reactive oxygen species (ROS)] to hydrogen peroxide. Hydrogen peroxide can subsequently be reduced to water by other antioxidant enzymes (Johnson et al. 1992) such as catalase, which is also a copper-containing enzyme. Other cuproenzymes and proteins that bind Cu are presented in Table 1 below.

**Table 1:** **Functions of established cuproenzymes and copper-binding proteins**^ɠ^

Copper enzyme/binding protein	Function
Cuproenzymes	
Amine oxidases (monoamine oxidase, tryamine oxidase and histamine oxidase)	Deamination of primary amines
Caeruloplasmin	Oxidation of iron, copper transport, antioxidant
Copper–zinc-superoxide dismutase	Free radical detoxification
Cytochrome c oxidase	Electron transport
Diamine oxidase	Oxidative deamination of amines
Dopamine b-hydroxylase	Catecholamine production
Extracellular superoxide dismutase	Free radical detoxification
Ferroxidase II	Oxidation of iron
Hephaestin	Copper oxidase, export of iron from intestine
Lysyl oxidase	Cross-linking of collagen and elastin
Peptidylglycine a-amidating monooxygenase	a-Amidation of peptides
Thiol oxidase	Disulfide bond formation
Tyrosinase	Melanin production
Intracellular copper transporters	
ATP7A (Menke’s disease protein)	Copper transporter
ATP7B (Wilson’s disease protein)	Copper transporter
Cox17	Copper chaperone for cytochrome c oxidase
CCS	Copper chaperone for superoxide dismutase
ATOX-1 (HAH1)	Copper homeostasis and antioxidant defence
Extracellular copper transporters	
Albumin	Copper transporter
Transcuprein	Copper transporter
Copper proteins with no known catalytic/transport function	
Clotting factor V	Blood clotting
Clotting factor VIII	Blood clotting
Metallothionein	Copper storage

^ɠ^Adapted from Bonham et al.(2002).

Because copper is involved in many functions of the body, its deficiency results in a range of symptoms. These symptoms include anaemia that does not respond to iron therapy but to copper supplementation. This anaemia is thought to result from defective iron mobilization due to decreased caeruloplasmin activity (Linus Pauling Institute 2007). Caeruloplasmin is the major copper-carrying protein in the blood, and also plays a role in iron metabolism. Other symptoms of copper deficiency are blood vessels that rupture easily, osteoporosis, joint problems, brain disturbances, hypopigmentation, impaired growth, increased susceptibility to infections due to poor immune function, among several others. Poor immune function is as a result of decreased number of white blood cells known as neutrophils, a condition known as neutropoenia (Castillo-Duran et al. 1983; Okoye 1992).

Large doses of copper, as in copper supplementation, are known to interfere with intestinal absorption of zinc and may be a contributing factor to zinc deficiency (World’s Healthiest Foods 2009). Zinc is an important component of biomembranes and an essential cofactor in a variety of enzymes (Soylak et al. 2001). Copper is found in many foods, particularly vegetable proteins such as nuts, beans, seeds and grains. Meats contain copper, but it is balanced by zinc which competes for its absorption. Chocolate is high in copper. A desire for copper may help explain chocolate cravings (Wilson 2011). According to national surveys, the average dietary intake of copper in the U.S. is approximately 1.0 to 1.1 mg/day for adult women and 1.2 to 1.6 mg/day for adult men (FNB 2001). Many diets, however, fail to provide this amount (Bonham et al. 2002). Over 30% of diets in USA and Europe provide less than 1.0 mg Cu/day (Klevay et al. 1993). In Nigeria, there have not been any researches on the copper content of local foodstuffs. The current survey by the Nigerian Food Consumption and Nutrition Survey 2001-2003 (Maziya-Dixon et al. 2004) did not include copper among the micronutrients surveyed. More so, that survey did not cover Plateau State of which Kanam Local Government Area is located where marginal zinc deficiency is suspected to be prevalent (ongoing PhD research in the University of Jos, Nigeria). Therefore, this research was aimed to determine the amount of copper in the local foodstuffs from Kanam Local Government Area in Nigeria with a view to knowing whether it could meet the daily dietary intake of copper, and whether it is high enough to interfere with zinc absorption.

## Materials and methods

### Samples collection

Samples of grains and nuts making up the diet of the subjects were collected randomly from the area of survey in July, 2011. Samples of plant materials were taken to the Taxonomy Unit of the Department of Plant Science and Technology, University of Jos, for identification, and were identified by Professor O.A.T. Namo as:

- Maize (yellow and white varieties) *Zea mays*
- Guinea Corn (red and white varieties) *Sorghum bicolor*
- Millet *Pennisetum glaucum*
- Common beans (cowpea) *Phaseolus vulgaris*
- Groundnut *Arachis hypogea*

### Sample preparation

All sample handling was performed wearing disposable, powder-free, latex gloves in clean areas. A combination of wet and dry ashing technique, adapted (with modification) from Hill et al. (1986), was used in the preparation of the food samples.

Fifty-one food samples (comprising of grains and nuts) were ground and homogenized in a stainless steel household food mill for 5 minutes (particle size > 300 μm). Sample masses ranging from 0.192 to 0.223 g (average 0.208 g) were weighed on Denver Instrument M–310 (Aldinger Company, USA) weighing balance. These were digested using a diluted oxidant mixture (1 ml deionised H_2_O + 0.5 ml double-distilled HNO_3_). The samples, in 13 × 100 mm borosilicate tubes were then placed in a heating block (Isotemp Dry Bath 145, Fisher Scientific Inc., Bohemia, NY, USA) and hydrogen peroxide (0.5 ml) was added to each to complete the first step of the digestion process. The operating temperature for this step was 95°C. At the end of this initial digestion, almost all the samples gave a black mass residue. The tubes are then placed upright in inverted 1000 ml glass beaker, covered with a watch glass (Plate 5), and placed in a muffled furnace (Ashing Oven Lindberg, USA).

Furnace temperature was set to increase from an initial 100°C to 375°C. The temperature was held at 375°C for 48 h. Thereafter, it decreased at the same rate as it was increased until it reached room temperature. After cooling, samples were removed from the furnace and 0.20 ml of deionised water [obtained from a Milli–Q water purification system (Millipore, Belford, MA, USA)] and 0.20 ml of double-distilled nitric acid (GFS Chemicals, Inc., Colunbus, OH, USA) were added to each.

Tubes were, again, placed in heating blocks (Isotemp Dry Bath 145, Fisher Scientific Inc., Bohemia, NY, USA) and the temperature was raised to 90°C first and then, 95°C. Hydrogen peroxide (50%) was added in 0.10 ml aliquots at 10–15 minutes interval until all black carbon particles were digested. Samples were allowed to evaporate to dryness and cooled. A white ash was obtained from each sample and 1.8 ml of deionised water and 0.2 ml of 1% double–distilled nitric acid were added to each (presumably one could use HCl in situations where nitrate ion is undesirable). Samples were reheated at 90°C for 15 minutes to dissolve the residue (ash) and cooled. From this solution, 0.1 ml was taken in 10 ml polypropylene tubes and made up to 5.0 ml by adding the following:

- 0.01 ml of 1:10 gallium, Ga, solution as internal standard;
- 4.89 ml of 0.1% double-distilled nitric acid.

Each tube was shaken on a Vortex (Vortex Genie 2™, Fisher Scientific Inc., Bohemia, NY, USA) and then, immediately used for the ICP-MS.

### ICP-MS instrumentation

A Perkin Elmer SCIEX^TM^ ELAN® 9000 ICP-MS (Norwalk, CT, U.S.A.) was used for the analysis of the samples described in this work.

Concentrations of copper was determined by inductively coupled plasma-mass spectrophotometry (ICP-MS) using internal standardisation with gallium in 2% HNO_3_ (Perkin Elmer Life and Analytical Sciences, Shelton, CT, U.S.A.) added at 0.1 μg/l concentrations to all measuring solutions for the correction of matrix effects. We conducted this analysis using ICP-MS because it was suitable for the analysis of low concentrations without large interference (Choi et al. 2009). The ICP-MS was operated at 1,400 W forward power with a coolant flow rate of 13.5 l/min, nebulizer gas flow rate of 1.15 l/min with concentric nebulizer. Spray chamber temperature was 4°C with cyclonic chamber. Sample delay and rinse times were 45 s with single reading. Sample uptake rate was 40 rpm.

The samples were analyzed at the Trace Mineral Laboratory of the Department of Nutritional Sciences, College of Human Sciences, Oklahoma State University, USA.

### Statistical analysis

Statistical analysis was performed using the computer software, SPSS Version 17.0 (SPSS Inc., Chicago). The statistical programme was SPSS Statistics Data Editor. Data were presented as means and standard deviations. The student’s t-test was used to examine the copper content of grains and nuts in the region. The acceptable level of statistical significance for all tests was p < 0.05. Results are expressed as arithmetic means ± standard deviation-SD.

## Results and discussion

The results of the analysis are as shown in the table below:

As can be seen from Table 2, there is, for each crop, a wide variation in copper content. However, the wide variations are statistically significant (p < 0.05) only in respect of the same varieties grown in the U.S.A. (USDA 2011). Both in terms of lowest and highest copper level detected and the mean values, cowpea (*Vigna unguiculata*) has the highest followed by groundnut (*Arachis hypogea*), while white maize (*Zea mays*) has the lowest copper content followed by yellow maize. In each case, the difference is statistically significant (p < 0.05). So, among the staple foodstuffs of the areas sampled, cowpea appears to be the richest source of dietary copper (based on copper content values) followed by groundnut while the two maize varieties are the poorest, in that order.

**Table 2:** Copper content of grains and nuts (μg/g) dry weight*

	Sample areas									
Sample	1	2	3	4	5	6	7	8	9	Mean ± SD
White Maize	1.43	2.35	4.04	2.69	2.04	1.53	1.74	1.38	2.73	2.22 ± 0.86
yellow Maize	4.43	1.24	1.39	2.73	1.62	2.36	1.23	2.11	2.96	2.35 ± 1.04
White Sorghum	3.44	2.79	3.70	4.58	3.40	3.26	4.10	3.39	3.38	3.62 ± 0.65
Red Sorghum	2.66	2.98	3.21	3.39	3.41	2.96	3.89	2.22	3.71	3.16 ± 0.52
Millet	5.10	3.95	4.12	2.48	4.31	4.07	3.62	3.73	3.56	3.83 ± 0.60
Groundnuts	9.50	8.29	10.03	6.26	9.74	11.81	8.32	8.47	6.16	8.73 ± 1.80
Cowpea	9.47	8.41	7.41	16.95	9.42	10.11	7.43	10.40	9.82	9.93 ± 2.85

*Tabulated Results are means of 3 determinations per sample area.

Studies on trace minerals, such as evaluation of Cu levels in Nigerian foods are very limited. This led to insufficient database for the contents of trace minerals in foods. To solve this problem the International Institute of Tropical Agriculture, IITA, conducted a survey between 2001–2003 (Klevay et al. 1993) to create awareness on the micronutrient deficiencies in Nigeria with emphasis on the trace elements zinc, iron and iodine, and the vitamins, A, D among others. The composition table of the United states Department of Agriculture (USDA 2011), provides a comprehensive database for trace elements and vitamins. But mineral contents of foods differ per region, and the rate of mineral utilization is different according to ethnic eating pattern. Therefore, one cannot rely on foreign data to evaluate mineral (especially copper) contents of our foods.

This analysis was conducted using inductively coupled plasma-mass spectrophotometry, ICP-MS, because it has low level of detection (LOD) and high reproducibility and accuracy (Choi et al. 2009). This study suggests the values of copper contents in a limited region, on limited food items and by limited analytical method. Therefore, there are limitations to using it as a database to estimate daily intake quantities of copper. Introduction of new food varieties and use of fertilizers and agrochemicals in crop production, among other factors, lead to changes in food composition (Malavolta et al. 1997; Reilly 1991). For this reason, also due to development in analytical techniques involving more accurate and precise methods of analysis, it is necessary to periodically re-evaluating food composition (Ferreira et al. 2005). While there is still insufficient data available in Nigeria on the contents of copper in food items, this study aims to contribute towards promoting further studies on trace minerals. From the result, consumption of foods rich in the legumes, cowpea and groundnuts, will likely meet up the RDA for copper of 0.9 mg/day and will not exceed the upper tolerable limit, UL, of 10 mg/day. Therefore, it is unlikely that any zinc deficiency in this area be attributed to high copper intake.

## Conclusion

Higher copper levels were detected in foods that are known to contain high amounts of proteins such as groundnuts and cowpea. Copper content of groundnuts were (6.16 to 11.81 μg/g). In samples of cereal grains, the copper content varies from 1.23 μg/g in yellow maize, to as much as 5.10 μg/g in millet. The highest copper content of 16.95 μg/g was detected in cowpea. In order to completely implement a future database on the nutritional properties of copper, studies must be carried out to accumulate data to ascertain it reliability and then to select representative values.
